# Land-use types and soil chemical properties influence soil microbial communities in the semiarid Loess Plateau region in China

**DOI:** 10.1038/srep45289

**Published:** 2017-03-28

**Authors:** Qin Tian, Takeshi Taniguchi, Wei-Yu Shi, Guoqing Li, Norikazu Yamanaka, Sheng Du

**Affiliations:** 1 State Key Laboratory of Soil Erosion and Dryland Farming on Loess Plateau, Institute of Soil and Water Conservation, Chinese Academy of Sciences and Ministry of Water Resources, Yangling 712100, China; 2 Institute of Soil and Water Conservation, Northwest A&F University, Yangling 712100, China; 3 University of Chinese Academy of Sciences, Beijing 100049, China; 4 Arid Land Research Center, Tottori University, Tottori 680-0001, Japan; 5 School of Geographical Sciences, Southwest University, Chongqing 400715, China

## Abstract

Similar land-use types usually have similar soil properties, and, most likely, similar microbial communities. Here, we assessed whether land-use types or soil chemical properties are the primary drivers of soil microbial community composition, and how changes in one part of the ecosystem affect another. We applied Ion Torrent sequencing to the bacterial and fungal communities of five different land-use (vegetation) types in the Loess Plateau of China. We found that the overall trend of soil quality was natural forest > plantation > bare land. Dominant bacterial phyla consisted of *Proteobacteria* (42.35%), *Actinobacteria* (15.61%), *Acidobacteria* (13.32%), *Bacteroidetes* (8.43%), and *Gemmatimonadetes* (6.0%). The dominant fungi phyla were *Ascomycota* (40.39%), *Basidiomycota* (38.01%), and *Zygomycota* (16.86%). The results of Canonical Correspondence Analysis (CCA) and Redundancy Analysis (RDA) based on land-use types displayed groups according to the land-use types. Furthermore, the bacterial communities were mainly organized by soil organic carbon (SOC). The fungal communities were mainly related to available phosphorus (P). The results suggested that the changes of land use type generated changes in soil chemical properties, controlling the composition of microbial community in the semiarid Loess Plateau region. The microbial community could be an indicator for soil quality with respect to ecological restoration.

Land-use change is one of the main factors affecting the biodiversity and functioning of terrestrial ecosystems^1^. Soil-residing microorganisms are living parts of soil ecosystems and have complex interactions with their environment^2^. At an ecosystem scale, they can directly influence soil ecological processes and maintain soil stability^3^,^4^. Meanwhile, the properties of microbes are manipulated by environment conditions^5^. Diverse systems with dissimilar microbial communities are expected.

To remedy adverse impacts to ecological environments, vegetation restoration has been conducted on the Loess Plateau of China, leading to changes in land-use patterns and above- and below-ground environments. These fluctuations, in turn, have profound indirect and direct effects on soil microorganisms^6^,^7^. The differences of microbe community composition could signify that biogeography is driven by special soil chemical properties that fluctuate among land-use types^8^. Studies have indicated that changes in land-use type have great effects on microbial communities^9^,^10^. A growing body of evidence not only supports this finding but also identifies land-use change as one of the most important drivers affecting biodiversity in terrestrial ecosystems^1^,^11^. However, in some ecosystems, soil chemical properties outweigh the effects of land-use type^12^. Furthermore, soil can release considerable CO_2_, even in the winter^13^ because of microbial activity. Although there have been several reports on soil microbial composition in the Loess Plateau of China^14^,^15^,^16^, the effects of land-use types and soil chemical properties on bacteria and fungi community composition during both growing and dormant seasons are still poorly understood. The main drivers of microbial community structure remain unknown because of the complex ecosystems in this region. There is an obvious need to understand the consequences of land-use changes in order to predict ecosystem stability and ecological services in the Loess Plateau of China.

In this study, we used the Ion Torrent sequencing approach to explore the vast uncultivated soil microbes that are directly related to the effects of land-use change on soil chemical properties. This study investigated the effects of (1) land-use types, (2) seasonality, and (3) soil chemical properties on fungal and bacterial communities in five different land-use types including natural and plantation forests, shrub land, and bare land. We aimed to understand whether land-use types and/or soil chemical properties are the primary drivers of soil microbial community structure.

## Materials and Methods

### Study area and experimental site

The study was conducted at Mt. Gonglushan in the southern suburb of Yan’an City, Shaanxi Province, in the central part of Loess Plateau (36°25.40′N, 109°31.53′E, 1353 m a.s.l.). This area is a fragile semiarid ecosystem and it contains one of the largest global loess areas. The site is also in the ecological transition zone between forest and forest-steppe ecosystems. Indigenous forests are present in sites with relatively little human disturbance. The mean annual precipitation and air temperature were 504.7 mm and 10.1 °C, respectively, based on a 40-year average (1971–2010) recorded by the city meteorological station^13^. In this region, the growing season for deciduous species occurs from April to October^17^. Five land-use types representing natural and planted forests, and non-forest lands were selected for this study: (1) Liaodong oak (*Quercus liaotungensis*), the typical climax forest type, (2) oriental arborvitae (*Platycladus orientalis*) which occurs as a small stand, (3) shrub land including species of early lilac (*Syringa oblata*), rose (*Rosa hugonis*), and *Caragana microphylla*, (4) black locust (*Robinia pseudoacacia*), a fast-growing introduced species which contributes to major plantations in the region^14^, and (5) bare land which was abandoned decades ago. These different land-use types are separated by approximately hundreds of meters and have the same climate^13^. More details about the sampling sites were displayed in Table 1 and the supplementary figure.

### Collection and preparation of soil samples

Soil sampling was conducted in August 2013 and February 2014, capturing the growing season and the dormant period, respectively. In this region, the plant growing period contains months from April to October, with June–August being the summer and December–February being the winter. The land-use types and their seasonal abbreviations in this study are as follows: oak forest (WO and SO), oriental arborvitae forest (WA and SA), black locust plantation (WL and SL), shrub land (WS and SS), and bare land (WB and SB) in winter and summer, respectively. In each land-use type, three soil samples were collected from locations approximately 50 m apart, generally on upper, middle, and lower locations of the land slope. At each location, a mixture of the top 20 cm of soil was collected from five sampling points along a “z”-type curve, and a 1 kg sample was obtained by the quartering method. After the roots and debris were removed, the samples were brought to the laboratory and preserved in two conditions: (1) stored at −40 °C in the refrigerator for molecular analysis and (2) sieved through 2 mm mesh and kept field-moist in a cooler at 4 °C^18^ until analyses for soil biological properties were conducted shortly thereafter.

### DNA extraction

For each location, genomic DNA was extracted from a 0.3 g soil sample using the PowerSoil^®^ DNA Isolation Kit (MOBIO Laboratories, Carlsbad, CA, USA), according to the supplier’s recommended protocol.

### Depth-dependent sequence-based analysis of DNA

For bacteria, the V4 region in 16 S rRNA gene was examined after the Polymerase Chain Reaction (PCR) with primers of S-D-Arch-0519-a-S-15 (CAGCMGCCGCGGTAA) and S-D-Bact-0785-a-A-21 (GACTACHVGGGTATCTAATCC)^19^. For fungi, the ITS1 region was examined with the PCR product amplified by the nested PCR approach^20^ with the first PCR using the primers of ITS9mum (TGTACACACCGCCCGTCG)^21^ and LR3 (CCGTGTTTCAAGACGGG)^22^ and the second PCR using the primers of ITS1-F_KYO2 (TAGAGGAAGTAAAAGTCGTAA) and ITS2_KYO2 (TTYRCTRCGTTCTTCATC)^23^. The forward primers to amplify the final target region for Ion Torrent sequencing (S-D-Arch-0519-a-S-15 for bacteria and ITS1-F_KYO2 for fungi) were linked with the Ion Torrent specific adapters and Ion-Xpress barcode to distinguish the origin samples for each sequence. PCR amplification was performed in a 25.5 μL cocktail including 22.5 μL of Platinum PCR Super Mix High Fidelity (Invitrogen, CA, USA), 2.0 μL of template DNA, and 0.5 μL (10 μM) of forward and reverse primers.

The PCR for bacteria was performed using a Veriti thermal cycler (Applied Biosystems) with initial denaturation at 94 °C for 3 min, 35 cycles of 30 s at 94 °C for denaturation, 30 s at 50 °C for annealing, and 1 min at 68 °C for elongation. The thermal profile of the first PCR for fungi was initial denaturation at 94 °C for 3 min, 20 cycles of 30 s at 94 °C for denaturation, 30 s at 48 °C for annealing, and 1 min at 68 °C for elongation. The second PCR was performed using 2.0 μL of the first PCR product diluted 10 times as the temperate DNA with the following thermal conditions: initial denaturation at 94 °C for 3 min, 35 cycles of 30 s at 94 °C for denaturation, 30 s at 50 °C for annealing, and 1 min at 68 °C for elongation. The PCR was performed twice for each sample and the amplicons were visualized on 0.7% agarose gels stained with SYBR Green I (Molecular Probes, Eugene, OR, USA).

The successful amplicons were pooled, precipitated, resuspended with isopropanol, and then cleaned using the QIAquick Gel Extraction (Qiagen) and the Agencourt AMPure XP (Beckman Coulter, Inc., Brea, CA), according to the manufacturer’s instructions. Quantitate purified each PCR product with the Qubit dsDNA HS Assay Kit on a Qubit fluorometer 2.0 (Invitrogen, Carlsbad, CA, USA). The library mix was then prepared with an equal amount of DNA from each barcode sample. Size distributions and DNA concentrations were measured by Agilent 2100 (Agilent Technologies, Inc.) and diluted to 12 pM before the amplification and purification using Ion OneTouch 2 system with Ion PGM temperate OT2 400 Kit (Life Technology, Inc.). Sequencing was performed using Ion PGM sequencer with Ion PGM sequencing 400 Kit and a 316 V2 tip (Life Technology, Inc.).

### Data analysis

Sequence data was generated in a standard flowgram format (sff) of each barcode sample. First, the primer sequence was eliminated using Cutadapt ver.1.3^24^. The subsequent sequence analysis was performed with a quantitative insight into microbial ecology (QIIME) pipeline^25^. The sequences shorter than 200 bp for bacteria and 160 bp for fungi and the sequences with an error rate greater than 0.2 were removed. Quality sequences were then clustered into operational taxonomic units (OTUs) with USERCH^26^ at a 97% similarity level. For the representative sequences of OTUs, a chimera check was performed by UCHIME^27^. Then, each OTU was classified into a taxonomic group with the databases of Greengenes for bacteria^28^ and UNITE for fungi^29^.

To assess microbial diversity among samples in a comparable manner, a normalized dataset was used for subsequent analyses, and the lowest sequence number for all sample subsets (23,261 for bacterial and 4,579 for fungi) was randomly selected. The diversity and evenness indices (Shannon and Simpson) were calculated at genetic distances (D) of 0.03. Shannon emphasizes the richness component of diversity, and Simpson emphasizes the evenness component. For both bacteria and fungi, there were only a few sequences that could not be identified to the phylum level. However, rarefaction analysis showed that all rarefaction curves were approaching smoothness with the increase of read number. It is, therefore, reasonable to infer that the obtained sequences covered the majority of the entire microbial community.

A non-parametric (Kruskal-Wallis) test was applied to determine whether there were significant differences in diversity indices among land use types (*P* < 0.05). To determine the relationships between soil chemical properties and diversity indices, Pearson correlations were calculated. In order to test a significant shift in microbial community composition based on land-use types, Adonis was used. The length of gradient was calculated firstly with the Detrended Correspondence Analysis (DCA) to determine which model would be most suitable for correlation analysis^30^. According to the results of DCA, a liner model Redundancy Analysis (RDA) was used for bacterial community as direct gradient analysis; while a unimodal model Canonical Correspondence Analysis (CCA) was used for fungal community. Besides the application of RDA and CCA^31^ which showed general relationships between each microbial community and soil chemical properties, the envfit was applied to the significance test between soil chemical properties and microbial community composition using the vegan package of R v.3.1.3 project (R Development Core Team. 2015).

## Results

### Land-use and soil characteristics

Soil chemical properties varied with land-use type (Table 2). The alkaline soil in this area has a pH value ranging from 8.18 to 8.48. The bare land had higher pH and lower soil nutrient than did the vegetated types. The black locust had higher pH value, highest available K and lower nutrient concentrations than did the other vegetated types. The natural climax oak forest had the highest soil organic carbon (SOC) and total nitrogen (TN), available P.

### Soil bacterial community composition and abundance

**Figure 1:**
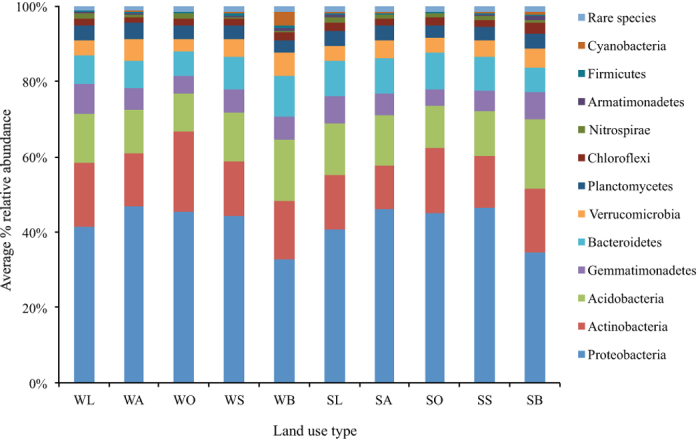
The relative abundances of major taxonomic groups at the phylum level for bacteria across all land-use types in winter and summer. Relative abundances are based on the proportional frequencies of bacterial DNA sequences that could be defined at the phylum. Site abbreviations starting with S and W indicate sampling in summer and winter, respectively. The land use types and their abbreviations are: oak forest (SO and WO), oriental arborvitae forest (SA and WA), black locust plantation (SL and WL), shrub land (SS and WS), and bare land (SB and WB) respectively. The figure is generated by using the R v.3.1.3 project (R Development Core Team. 2015).

The dominant bacterial phyla contained *Proteobacteria* (42.35%), *Actinobacteria* (15.61%), *Acidobacteria* (13.32%), *Bacteroidetes* (8.43%), and *Gemmatimonadetes* (6.0%) (Fig. 1). In addition, *Planctomycetes, Verrucomicrobia, Thaumarchaeota, Chloroflexi*, and *Nitrospirae* with low abundances (<5%) existed in most soils, and 29 other rare phyla were also distinguished. *Alphaproteobacteria* was the most abundant *Proteobacteria*, followed by *Deltaproteobacteria, Betaproteobacteria*, and *Gammaproteobacteria. Thermoleophilia, Actinobacteria*, and *Acidimicrobiia* were the most abundant groups of *Actinobacteria*. The relative abundance of *Proteobacteria* was greater in natural vegetation (oak forest, oriental arborvitae forest, and shrub land) than it was in the bare land and black locust; *Acidobacteria* showed a reverse trend. The dominant phyla were the same across land-use types, but their relative abundances were different.

### Relationships between soil chemical properties and bacteria distributions

**Figure 2:**
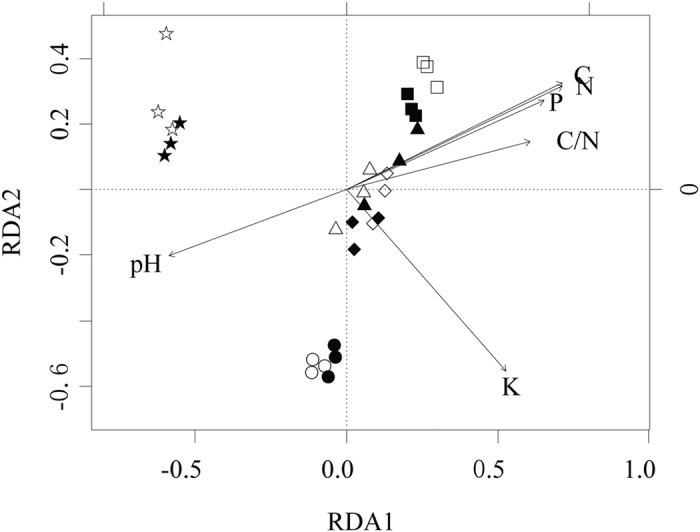
Redundancy analysis (RDA) of sampling sites and soil factors based on bacterial OTUs. The land-use types and their icons are as follows: black locust plantation (⦁ and ⚬), oriental arborvitae forest (◆ and ♢), oak forest (◼ and ◻), shrub land (▴ and ▵), and bare land (★ and ☆), in summer and winter respectively. The figure is generated by using the R v.3.1.3 project (R Development Core Team. 2015).

The results of the RDA of soil chemical properties and bacterial community composition showed that groups according to land-use types (Fig. 2). Bare land and black locust were grouped into clusters distinct from others along the first axis comprising 24.78% of the total variation of site-environment relation. The second axes comprised 6.37% of the total variation and separated black locust from bare land and oak forest. Thus the bare land, black locust as well as others were grouped into distinct clusters, concurring with the Adonis results (*P* < 0.01). Soil chemical properties account for variation in bacterial community. Each index of soil chemical properties showed significant association with bacterial community composition (*P* < 0.001). The main factor for grouping the bare land sites was pH with higher abundance. SOC, TN, available P, and C/N tended to have lower values and were negatively associated with the bare land but positively associated with the oak forest sites, while the black locust sites were grouped with higher available K. In total, SOC exerted the largest effect on the distribution of bacterial communities.

For bacterial, there were diversity (Shannon) and evenness (Simpson) differences between black locust and bare land in the summer, but others has no difference (*P* < 0.05) (Table 3). The diversity indices showed significant correlation with soil K (*R*^*2*^ = 0.73, *P *<* *0.001; *R*^*2*^ = 0.59, *P *<* *0.001, respectively).

### Soil fungi community composition and abundance

**Figure 3:**
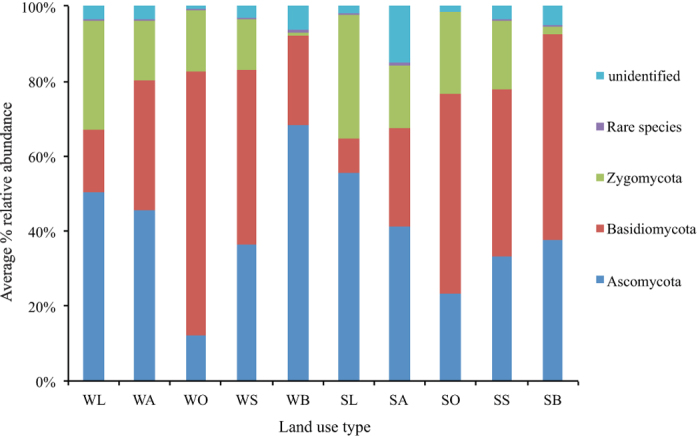
The relative abundances of major taxonomic groups at the phylum level for fungi across five land-use types in winter and summer. The land use types and their abbreviations are: oak forest (SO and WO), oriental arborvitae forest (SA and WA), black locust plantation (SL and WL), shrub land (SS and WS), and bare land (SB and WB) respectively. The figure is performed in R v.3.1.3 project (R Development Core Team. 2015), using the R packages vegan.

The majority of dominant fungi phyla (Fig. 3) belonged to *Ascomycota* (40.39%), *Basidiomycota* (38.01%), and *Zygomycota* (16.86%). *Sordariomycetes, Dothideomycetes*, and *Lecanoromycetes* were the most abundant groups of *Ascomycota. Agaricomycetes* and *Tremellomycetes* were the most abundant groups of *Basidiomycota*. Most of the *Zygomycota* were incertae. *Ascomycota* was the most abundant in black locust, and bare land (but summer bare land samples), but was least abundant in the oak forest. The oak forest had the most *Basidiomycota*, while the black locust had the least. There were no differences in fungi diversity indices between land-use types (Table 3) and no correlations with soil chemical properties.

### Relationships between soil properties and fungi community distribution

**Figure 4:**
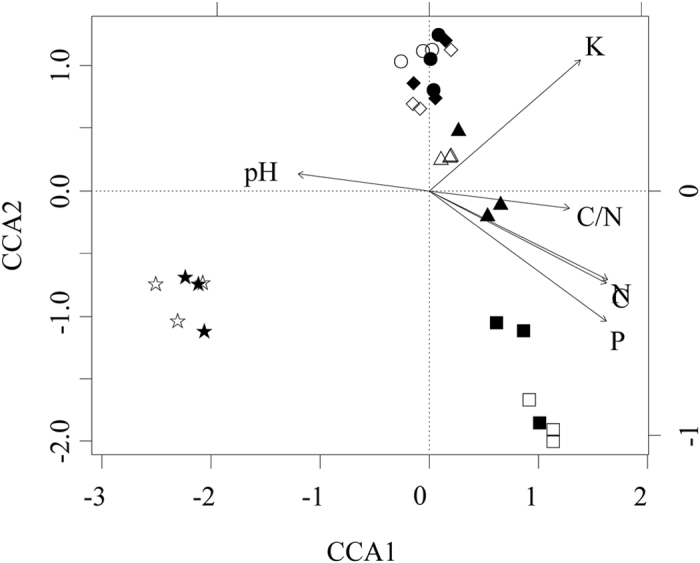
Canonical correspondence analysis (CCA) of sampling sites and soil factors based on Hellinger-transformed fungi OTUs. The land-use types and their icons are as follows: black locust plantation (⦁ and ⚬), oriental arborvitae forest (◆ and ♢), oak forest (◼ and ◻), shrub land (▴ and ▵), and bare land (★ and ☆), in summer and winter respectively. The figure is performed in R v.3.1.3 project (R Development Core Team. 2015), using the R packages vegan.

From the CCA with all the soil chemical properties, the fungal community under the similar land use types typically clustered together (Fig. 4). The bare land type was clustered far away from other land-use types along the first axis comprising 8.38% of the total variation. The oak forest type and bare land type clustered in different groups from the black locust type and oriental arborvitae forest type along the second axes comprising 6.01% of the total variation. The Adonis results also demonstrated those appearances (*P* < 0.01). Meanwhile, soil chemical properties exerted a significant effect on the composition of fungal community. SOC, total N, available K and P showed significant association with fungal community composition (*P* < 0.001), and the significance of soil pH and C/N is 0.01. The main grouping factor for the bare land type was pH, and the oak forest type was clustered in the area with high SOC, TN, P, and C/N. Available K showed a positive association and higher abundance in the black locust. In total, available P content was the main factor influencing the fungi community distribution.

## Discussion

A previous study showed that similar land-use types have similar soil chemical properties^9^. We found that vegetated land had advantages in soil chemical properties over bare land and that the natural forest land had advantages over the other land-use types. These findings demonstrated the potential positive effects of vegetation restoration, especially with natural vegetation types, on soil nutrient conditions. The bare land and black locust types had higher pH values than did the other land-use types, illustrating that plantations do not improve soil conditions to the same extent as did natural vegetation types. Therefore, reforestation projects, and the resulting differences in land-use type, might contribute to dissimilar microenvironments, which are closely associated with soil-borne microbes.

Similar to the results of early surveys^6^,^32^, the dominant bacterial phyla in Loess Plateau samples were *Proteobacteria, Acidobacteria, Actinobacteria*, and *Bacteroidetes*. This is different from the results of Xiong, *et al*.^33^ who showed that *Bacteroidetes* and *Firmicutes* were dominant in alkaline lake sediments.

Microbial community structure was driven by both land-use type and soil chemical properties. The importance of soil chemical properties in shaping microbial communities has been established by a number of studies. As general rule, soil pH plays a key role in controlling the distribution of microbial communities^34^,^35^,^36^. In our study, although pH can significantly structure bacterial communities, the main factor that influenced bacterial community structure was SOC. Our results agreed with the results of other studies that showed that SOC had the greatest effect on soil bacterial community structure^37^,^38^. This may be reflected by the fact that the high pH soils in the bare land and black locust forest types were nutrient-poor and therefore grouped distinctly. Variances in soil quality properties and the copiotrophic–oligotrophic classification of soil bacterial phyla might also explain this inconsistency^37^. Similar to Zhang, *et al*.^39^, we found that the relative abundance of *Acidobacteria* increased with soil pH. However, other studies showed that the relative abundance of *Acidobacteria* was typically negatively correlated with soil pH^35^,^36^,^40^. Other studies indicated that the relative abundance of *Acidobacteria* only started to decrease markedly when the pH was below 5.5^35^,^36^,^41^. In this study, soil pH had a narrow alkaline range (8.18 to 8.48).

The dominant bacteria (*Proteobacteria, Acidobacteria*, and *Actinobacteria*) were greatly responsive to SOC content. The results obtained in this study are in line with the general framework that uses the terms copiotroph and oligotroph to describe microbial communities with ecological attributes typical of *r*- and *K-* strategists, respectively^37^. Copiotrophs require high nutrition and can utilize labile SOC to keep growth high rates if resource conditions are sufficient. For example, in this study, *Proteobacteria* increased with SOC and were the most abundant in SOC-rich oak forest soils. On the contrary, there were many more oligotrophic groups of *Acidobacteria*, which is expected to be the first bacteria to colonize nutrient-poor substrates in areas with low resource availability^37^, such as bare land and black locust forests. Other studies have shown similar results in that *Acidobacteria* are less abundant and *Proteobacteria* are more abundant in soils with high resource availability^31^,^42^. Oligotrophs are probably able to outcompete copiotrophs under stress conditions of low resource concentrations^37^. Our results further confirmed this and extended evidence for these ecological classifications for direct or indirect bacterial responses to soil nutrient availability. Several other studies have also showed that the *Proteobacteria/Acidobacteria* ratio reflected the soil chemical properties, with lower ratios occurring in oligotrophic soils. Our study further supports this finding because we found the highest ratios in the oak forest site and the lowest ratios in bare land and black locust forest. Therefore, it is likely that land-use and soil nutrient conditions are the major explanatory variables of bacterial community structure. In addition, bacterial community structure can also reflect the soil nutrient status in Loess Plateau, China.

We found that the fungal taxa mainly belonged to *Ascomycota, Basidiomycota*, and *Zygomycota*, consistent with previous studies^43^,^44^. However, different results were reported for Arctic soils where *Zygomycota* and *Chytridiomycota* dominated^45^,^46^. *Ascomycota* and *Basidiomycota* tended to reside in cooler and arid environments because of their evolutionary histories^47^.

Fungal communities had lower diversity than did bacterial communities, and there was no correlation with the soil chemical properties. In contrast to the bacterial communities, the fungal communities were mainly driven by soil P content, consistent with results from Lauber *et al*.^10^, which demonstrated that extractable P concentrations were often correlated with fungal communities. This further suggests that bacterial and fungal communities respond to different soil chemical properties factors.

Leckie *et al*.^48^ and Kasel *et al*.^48^,^49^ determined that land-use played a role in modulating the soil fungal community structure. Lauber *et al*.^10^ observed that soil chemical properties more strongly influenced fungal communities than did land-use type. In this study, the CCA showed that the bare land, black locust forest and oak forest were grouped into distinct clusters according to land-use types, and the primary controlling factor was available P. Sites with similar available P content tended to cluster together. This may be related to the corresponding different microclimates for each land-use type, or a function of the fungi. Distribution of fungi was related to soil P. *Basidiomycota* abundance increased with increasing soil P, but *Ascomycota* abundance decreased. Oak trees serve as host species and the relatively P-rich oak forest sites contained more *Basidiomycota. Basidiomycota* is the dominant ectomycorrhizal species and is conducive to the success of reforestation projects. In contrast, Lauber^10^ found that P-rich soils contained a higher content of *Ascomycota* than *Basidiomycota*. The reason for this discrepancy may be related to the range of soil P (1.8–17 mg·kg^−1^ in Lauber^10^ and 1.65–3.31 mg·kg^−1^ in our study). *Zygomycota*, as saprotrophic species, are responsible for decomposing plant litter, alter soil chemical properties^43^,^50^. This was mirrored by most of the differences between the dystrophic bare land site and the other forest sites. There was much more litter in forests than there was in the bare land area. There were more Ascomycota in the bare land and black locust, corresponding with the report that approximately 46% Ascomycota can form lichen which can grow and persist in deserts and mountaintops^51^. New microbial communities were forced to form so as to adapt to the hazardous environment^52^. This was confirmed by our study results, which showed that bare land and black locust have a large number of lichen. Without vegetative cover, bare land becomes more reflective and is often subjected to direct sunlight and subsequent drought. Lichens form poikilohydric biomes, meaning they desiccate and remain dormant and highly tolerant of dry conditions, but can rehydrate after rain. We can therefore infer that different land-use types effect the selection of fungal types in terms of those that are the most fit for survival in the given conditions. This can explain the finding that vegetated land and natural forests had advantages in soil quality than did bare land and planted forests, respectively.

While seasonal changes in microbial community structure have been noted^53^,^54^, we expected the seasonal differences in the microbial communities to reflect the vitality of the summer samples and the dormancy of the winter samples. However, we found that seasonal variations were actually relatively small. Independent of season, the microbial communities from the same site was clustered together. This is possibly because these communities were uniform in their responses to seasonal change with little change in cellular abundance from physiological adaptation^55^,^56^. Seasonal variations in many fungal dynamics are in large part tied to plant species^57^,^58^. In addition, under repeated stresses, microbial communities may become more resistant or possess conservative strategies^59^,^60^. This study region experienced repeated drought and heavy rainfall stresses. In addition, another possible reason was that our DNA-based analyses included a potentially huge dormant community or even dead microbial community^61^.

Jangid, *et al*.^55^ showed that nutrient input had a larger effect on microbial structure than did season. In this study, the fact that soil microbial community structure correlated with the soil resources suggests that the microbial community may be more responsive to changes in resource inputs. Although seasonal change can also affect C inputs into the soil, samples were taken from non-rhizosphere soil. Under different land-use types, soil properties might be relatively stable and be able to support microbial communities with similar resistance and restorative properties.

In conclusion, similar land-use types usually have similar soil chemical properties and, similar microbial communities in a specific area. Our results support previous studies that soil chemical properties and land use types have crucial influence on the composition and divertity of microbial community. The changes of land use type generated soil chemical properties, both of which played a significant role in controling the composition of microbial community. In turn, the microbial community composition might be an indicator of changes in soil chemical properties or responses to land use types after ecological restoration on the Loess Plateau.

## Additional Information

**How to cite this article**: Tian, Q. *et al*. Land-use types and soil chemical properties influence soil microbial communities in the semiarid Loess Plateau region in China. *Sci. Rep.* **7**, 45289; doi: 10.1038/srep45289 (2017).

**Publisher's note:** Springer Nature remains neutral with regard to jurisdictional claims in published maps and institutional affiliations.

## Supplementary Material

Supplementary Dataset

## Figures and Tables

**Table 1:** The information of sampling site for different land-use types.

Sites	Type	Slope (°)	Tree coverage (%)	Age (year)
Black locust	Planted forest	26	60	30
Oriental arborvitae	Non-typical natural forest	10	35	50
Liaodong oak	Secondary natural forest	22	75	60
Shrub land	Mixed natural shrub	10	80	>60
Bare land	Un-forested	10	0	30

**Table 2:** Soil chemical properties for different land-use types.

Sites	SOC	TN	Available Potassium (K)	Available Potassium (P)	pH	C/N
	(%)	(%)	(mg·kg^−1^)	(mg·kg^−1^)		
SL	1.08 ± 0.09 c	0.11 ± 0.00 c	190.13 ± 10.67 a	2.12 ± 0.07 b	8.45 ± 0.04 a	9.51 ± 0.50 b
SA	1.88 ± 0.24 b	0.17 ± 0.02 b	139.73 ± 33.94 b	2.10 ± 0.17 b	8.18 ± 0.06 b	11.08 ± 0.15 a
SO	2.86 ± 0.42 a	0.26 ± 0.02 a	142.88 ± 7.76 b	3.31 ± 0.35 a	8.23 ± 0.11 b	11.07 ± 0.93 a
SS	2.26 ± 0.27 b	0.23 ± 0.02 a	174.17 ± 14.45 ab	2.46 ± 0.12 b	8.27 ± 0.03 b	9.84 ± 0.23 ab
SB	0.70 ± 0.09 c	0.08 ± 0.01 d	76.22 ± 7.11c	1.65 ± 0.05 c	8.48 ± 0.12 a	8.94 ± 0.59 b

Site abbreviations are as follows: SL: black locust plantation; SA: oriental arborvitae forest; SO: oak forest; SS: shrub land; SB: bare land. Abbreviations starting with S indicate summer samples. Data are expressed as mean ± standard errors. Different letters after the values indicate treatments with significant differences (*P* < 0.05).

**Table 3:** Diversity indices for different land-use types.

Diversity indices	Bacterial Shannon	Bacterial Simpson	Fungal Shannon	Fungal Simpson
WL	9.51 ± 0.03 ab	0.10 ± 0 a	5.14 ± 0.46 a	0.94 ± 0.02a
WA	9.31 ± 0.08 ab	0.10 ± 0 a	5.16 ± 0.80 a	0.93 ± 0.05 a
WO	9.32 ± 0.04 ab	0.10 ± 0 a	4.41 ± 0.15 a	0.85 ± 0 a
WS	9.36 ± 0.10 ab	0.10 ± 0 a	4.12 ± 2.09 a	0.78 ± 0.25 a
WB	8.89 ± 0.53 ab	0.99 ± 0.01 a	4.76 ± 0.64 a	0.92 ± 0.04 a
SL	9.55 ± 0.01 a	0.10 ± 0 a	4.85 ± 0.10 a	0.92 ± 0.00 a
SA	9.32 ± 0.08 ab	0.10 ± 0 a	4.98 ± 0.42 a	0.93 ± 0.04 a
SO	9.31 ± 0.04 ab	0.10 ± 0 a	5.18 ± 1.07 a	0.91 ± 0.09 a
SS	9.38 ± 0.02 ab	0.10 ± 0 a	5.18 ± 1.33 a	0.87 ± 0.16 a
SB	9.10 ± 0.20 b	0.10 ± 0 a	3.91 ± 1.31 a	0.77 ± 0.18 a

The land-use types and their seasonal abbreviations are: black locust plantation (WL and SL), oriental arborvitae forest (WA and SA), oak forest (WO and SO), shrub land (WS and SS), and bare land (WB and SB) in winter and summer, respectively. Data are expressed as mean ± standard errors. Different letters after the values indicate significant differences (*P* < 0.05).
