# Supplementary material for: Land-use types and soil chemical properties influence soil microbial communities in the semiarid Loess Plateau region in China
Source: Sci Rep. 2017 Mar 28;7:45289. doi: 10.1038/srep45289 (PMC5368647; doi:10.1038/srep45289)
Supplement: Supplementary Dataset [file srep45289-s1.doc]

**Scientific Reports - Electronic Supplementary Material**

**Land-use types and soil chemical properties influence soil microbial communities in the semiarid Loess Plateau region in China**

Qin Tian^1,2,5^, Takeshi Taniguchi^3^, Wei-Yu Shi^4^, Guoqing Li^1,2^, Norikazu Yamanaka^3^, Sheng Du^1,2,*^

1 State Key Laboratory of Soil Erosion and Dryland Farming on Loess Plateau, Institute of Soil and Water Conservation, Chinese Academy of Sciences and Ministry of Water Resources, Yangling 712100, China

2 Institute of Soil and Water Conservation, Northwest A&F University, Yangling 712100, China

3 Arid Land Research Center, Tottori University, Tottori 680-0001, Japan

4 School of Geographical Sciences, Southwest University, Chongqing 400715, China

5 University of Chinese Academy of Sciences, Beijing 100049, China

Corresponding Author: Sheng Du

State Key Laboratory of Soil Erosion and Dryland Farming on the Loess Plateau, Institute of Soil and Water Conservation, Chinese Academy of Sciences and Ministry of Water Resources, Yangling 712100, China

Email: [shengdu@ms.iswc.ac.cn](mailto:shengdu@ms.iswc.ac.cn)

Tel.: +86-29-87012411

Fax: +86-29-87012210


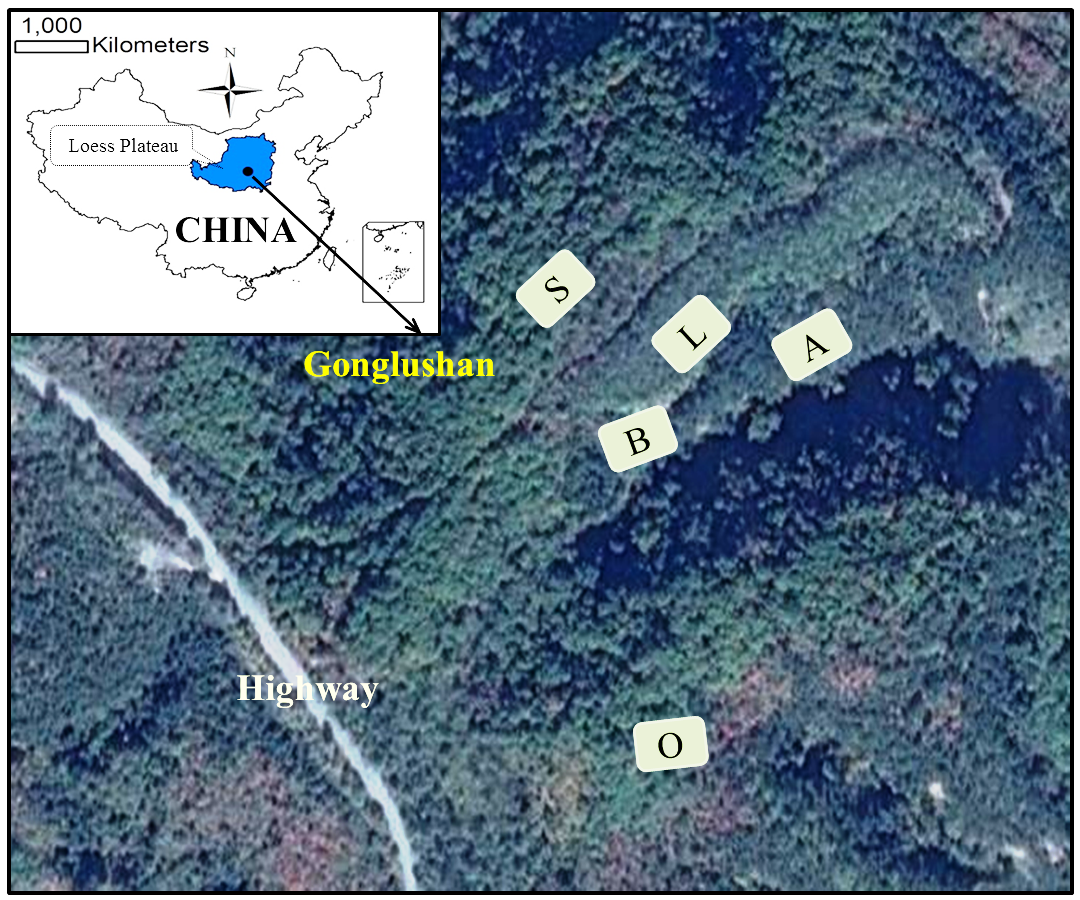


**Figure S1.**Location of the study site, Mt. Gonglushan in the central part of Loess Plateau in northern China (36°25.40′N, 109°31.53′E, 1353 m a.s.l.), and the five sampling places for different land-use types. Abbreviations are as follows: L: black locust plantation; A: oriental arborvitae forest; O: oak forest; S: shrub land; and B: bare land. The China map illustrating the location of Loess Plateau was created using the tool of ArcGIS 9.3 (ESRI, Redlands, CA, USA, http://www.esri.com/). The image for study site of Gonglushan was adopted from online version of Google map (Imagery ©2017 CNES / Astrium, Map data ©2017. Accessed on 16 Jan 2017; http://www.google.com/maps).
